# RAMPing up knowledge of the translocon

**DOI:** 10.7554/eLife.98548

**Published:** 2024-05-24

**Authors:** Dimitrios Vismpas, Friedrich Förster

**Affiliations:** 1 Bijvoet Center for Biomolecular Research, Utrecht University Utrecht Netherlands

**Keywords:** multipass translocon, cryo-EM, Sec61, RAMP4, ribosomes, None

## Abstract

Advanced cryo-EM approaches reveal surprising insights into the molecular structure that allows nascent proteins to be inserted into the membrane of the endoplasmic reticulum.

**Related research article** Lewis AJO, Zhong F, Keenan RJ, Hegde RS. 2024. Structural analysis of the dynamic ribosome-translocon complex. *eLife* **13**:RP95814. doi: 10.7554/eLife.95814.

The endoplasmic reticulum is a cellular compartment that helps to ready certain newly made proteins for their role in the organism. Membrane proteins, for example, first need to be inserted into the membrane of the reticulum before they can be modified and trafficked to their destination. Secretory proteins, on the other hand, are translocated across the reticulum membrane and into the compartment to be altered and prepared for export.

The translocon, an evolutionary conserved gate-like complex, ensures that both secretory and membrane proteins are translocated across or inserted in the reticulum membrane. In eukaryotes, the central component of the translocon – a membrane channel known as Sec61 – can physically associate with ribosomes in the midst of synthesizing proteins to ensure the seamless processing of nascent molecules (Rapoport et al., 2017). Highly secreting cells such as those in the pancreas, for instance, often feature large numbers of ribosomes bound via Sec61 to vast amounts of endoplasmic reticulum membranes (Palade, 1975).

To cope with processing many different proteins, Sec61 teams up with other molecular actors in a dynamic fashion (Gemmer and Förster, 2020). Technological advances have provided important insights into these highly complex structures and interactions. While electron microscopy has always been central to these studies (Palade, 1975), cryo-EM single particle analysis has made it possible to capture the different conformations of individual ribosome-translocon complexes in solution at increasingly higher resolution (Braunger et al., 2018; Voorhees et al., 2014). Closely examining the parts of the complex that show structural variability relies on analysing large numbers of images, which is now possible due to improvements in computational power. Similar analyses of cryo-electron tomography data, which provides three-dimensional modelling of molecular actors, have captured the ribosome-translocon complex in the native membrane environment (Gemmer et al., 2023). Finally, AI-driven advances in protein structure prediction have greatly supported the interpretation of fuzzier electron microscopy maps that had previously evaded molecular assignment.

Taken together, these studies show that Sec61 forms a channel which can open to let target molecules cross the membrane, enabling secretory proteins to be imported from the cytosol and into the reticulum. In addition, Sec61 features a lateral gate that participates in inserting nascent membrane proteins into the reticulum membrane; by opening directly into the lipid bilayer, this sideways exit helps hydrophobic domains to be transferred to the membrane as the proteins are processed by the translocon. Other factors associated with Sec61 have also been identified, such as TRAP, which helps with the early steps of the insertion of a protein through the channel, or RAMP4, whose role is still unknown (Görlich and Rapoport, 1993).

Previous work has also revealed that two types of translocons exist, one specializing in secretory proteins and the other in multipass transmembrane domains (Gemmer et al., 2023; McGilvray et al., 2020; Smalinskaitė et al., 2022; Sundaram et al., 2022). This multipass translocon recruits various cofactors at different stages, including the GEL complex, which inserts proteins into the reticulum membrane, and a chaperone complex, known as PAT, which assists in protecting and folding membrane proteins. However, it remains unclear exactly how this complex comes together, helps to insert proteins into the membrane via its lateral gate, or interacts with ribosomes. Now, in eLife, Ramanujan Hegde of the MRC Laboratory of Molecular Biology (LMB) and co-workers – including Aaron Lewis as first author – report new insights into these processes by applying advanced approaches and analyses to existing data (Lewis et al., 2024).

The team (who are based at the LMB and the University of Chicago) relied on a large cryo-EM single particle analysis dataset that captured solubilized ribosome-translocon complexes ‘stalled’ at a specific step during the synthesis and membrane insertion of Rho^ext^ (a construct carrying two transmembrane domains from the protein rhodopsin). Previous analyses had focused on only one of several types of ribosome-translocon complexes that can form at this stage – the fully-assembled multipass translocon, in which PAT is integrated and the lateral gate is closed (Figure 1A). In contrast, Lewis et al. examined PAT-less complexes, showing that three distinct intermediates can form as Rho^ext^ is processed. As opposed to the fully loaded multipass translocon, two of these structures (which the team then focuses on) feature an open Sec61 lateral gate that can interact with the membrane of the reticulum and therefore allow protein insertion.

**Figure 1.**
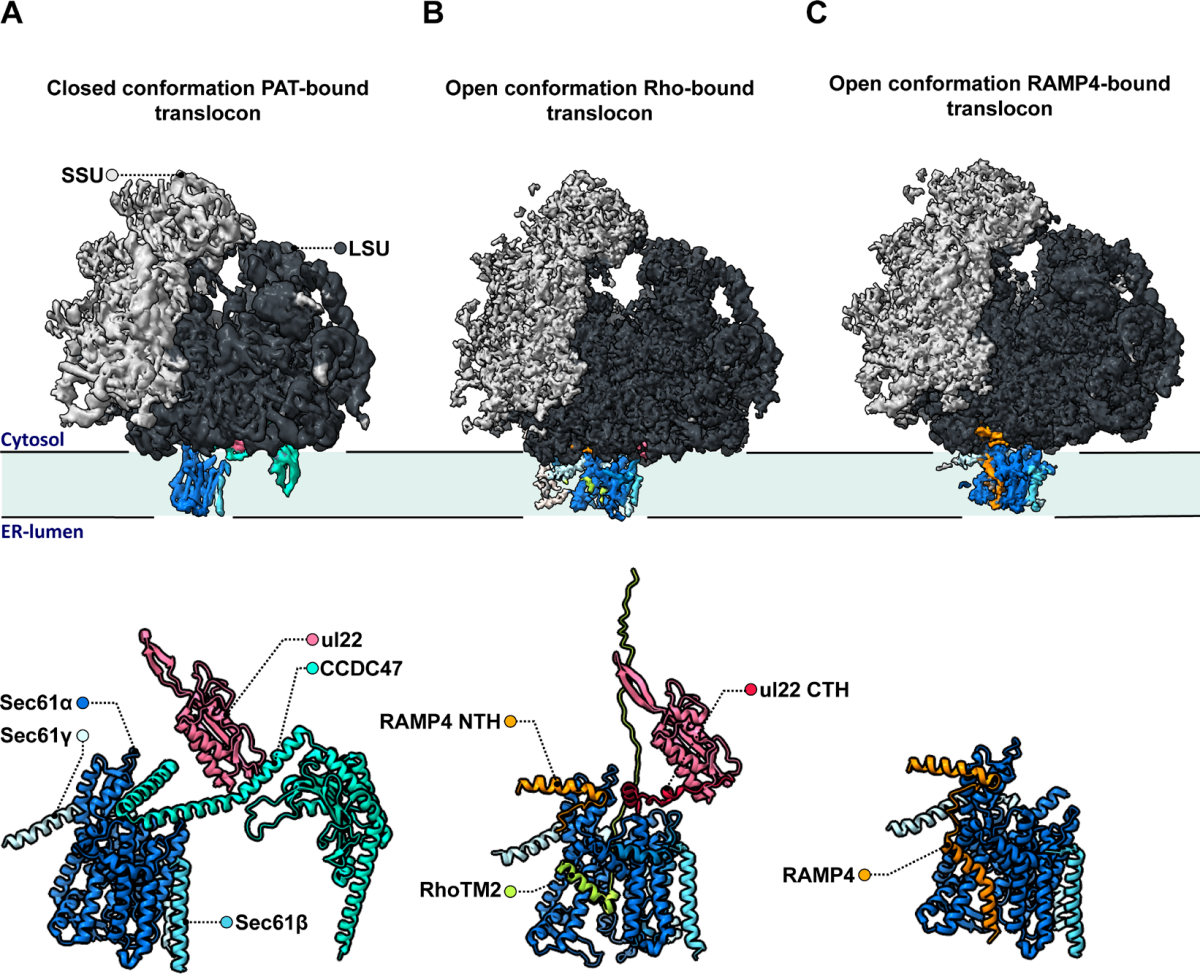
Molecular structures of the different intermediates of ribosome-bound multipass translocons forming during the biogenesis of Rho^ext^. Lewis et al. examined the structure of various intermediates of the multipass translocon as it processed a nascent Rho^ext^ construct emerging from ribosomes (small and large subunits in light and dark grey, respectively, with ribosomal protein uL22 in hot pink). This highly dynamic complex is formed of a Sec61 channel (light and dark blue) studded through the membrane of the endoplasmic reticulum, which can associate with various molecular actors. (**A**) In a fully loaded multipass translocon, Sec61 binds to the chaperone PAT, which connects to the ribosomal protein uL22 via its CCD47 subunit (aqua). In this conformation, the lateral gate (a sideway exit that allows the insertion of transmembrane domains into the membrane) is closed. (**B**) Top: In one type of PAT-less multipass translocon, the second transmembrane domain in the Rho^ext^ construct (RhoTM2; green) is bound to the lateral gate of Sec61, which is open. Bottom: Certain transmembrane segments of RAMP4 (orange) can get temporarily replaced by the hydrophobic domains of the protein processed by the translocon, such as RhoTM2 (green; here bound to Sec61). In addition, in the absence of CCDC47 the uL22 C-terminal helix (red) of the ribosome gets ordered and forms a lid blocking an escape route for nascent proteins to the cytosol. (**C**) In the other type of open PAT-less multipass translocon (in which the lateral gate is also open), RAMP4 (orange) binds to Sec61 and acts as a fourth subunit for the channel, potentially participating in its opening.

One of these intermediates is characterized by the presence of the second transmembrane rhodopsin domain at the lateral gate, while the first domain has already inserted into the membrane and is not resolved (Figure 1B, top). Such arrangement may be an alternative to the previously identified insertion mechanism for multipass membrane proteins via GEL.

However, a surprise emerged when the nature of the complex inhabiting the lateral gate of the second type of intermediate was analyzed. Lewis et al. showed that the transmembrane domain of RAMP4 binds at this location in a fashion similar to signal peptides (the short sequences that secretory proteins carry to direct them towards the reticulum). Meanwhile, its N-terminus attaches to the ribosome.

Based on comparisons with the structure of the translocon that processes secretory proteins, the team suggested that RAMP4 acts as a fourth subunit of Sec61, keeping the channel open and the complex tethered to ribosomes (Figure 1C). Some of its internal segments may get transiently displaced as transmembrane domains are inserted into the membrane and RAMP4 probably then dissociates from the complex when PAT is recruited.

Another unexpected finding is that the C-terminal helix of the ribosomal protein uL22, which is typically disordered in soluble ribosome structures, engages in a space between the ribosome and Sec61 in both types of intermediates (Figure 1B, bottom). The uL22 terminus, which Lewis et al. also localize in the secretory translocon upon re-analysis, blocks one of two possible escape routes for nascent proteins before they transfer into the Sec61 channel.

Overall, these discoveries allow many new hypotheses and functional studies to be designed. A short-term aim will be to validate the positioning of RAMP4 in the Sec61 lateral gate of secretory translocons, and whether it is the cause or the consequence of its opening. The longer-term aim will be to substantiate the precise biochemical functions and mechanisms of RAMP4 and the TRAP complex, which are physically closely associated. Deletion studies hint at TRAP and RAMP4 impacting secretory proteins, with their positioning near the Sec61 lateral gate strongly supporting their involvement in directing signal peptides to this location. Moreover, TRAP and RAMP4 are both induced upon stress (Nagasawa et al., 2007; Yamaguchi et al., 1999). The structural findings reported by Lewis et al. may be a decisive step towards understanding the precise function of RAMP4 and TRAP in protein biogenesis and endoplasmic reticulum stress.
